# Iodine nutrition status in Africa: potentially high prevalence of iodine deficiency in pregnancy even in countries classified as iodine sufficient

**DOI:** 10.1017/S1368980020002384

**Published:** 2021-08

**Authors:** Charles Bitamazire Businge, Benjamin Longo-Mbenza, Andre Pascal Kengne

**Affiliations:** 1 Department of Medicine, Faculty of Health Sciences, University of Cape Town, Cape Town, South Africa; 2 Department of Obstetrics and Gynaecology, Faculty of Health Sciences, Walter Sisulu University, Mthatha, South Africa; 3 Faculty of Medicine, University of Kinshasa, Kinshasa, Democratic Republic of Congo; 4 Lomo University of Research, Kinshasa, Democratic Republic of Congo; 5 Non-Communicable Research Unit, South African Medical Research Council, Cape Town, South Africa

**Keywords:** Iodine deficiency, Urinary iodine concentration, Pregnancy, Africa

## Abstract

**Objective:**

To assess the burden of iodine deficiency in pregnancy in Africa using estimated pregnancy median urinary iodine concentration (pMUIC).

**Design:**

pMUIC for each African country was estimated using a regression equation derived by correlating the school-age children (SAC) median UIC (mUIC) and pMUIC from countries around the globe, and the SAC mUIC data for African countries obtained from the Iodine Global Network (IGN) 2017 and 2019 Score cards.

**Setting:**

Iodine deficiency was endemic in many African countries before the introduction of iodine fortification, mainly through universal salt iodisation programmes about 25 years ago. There is a scarcity of data on the level of iodine nutrition in pregnancy in Africa. Women living in settings with pMUIC below 150 µg/l are at risk of iodine deficiency-related pregnancy complications.

**Participants:**

Fifty of the fifty-five African countries that had data on iodine nutrition status.

**Results:**

A cut-off school age mUIC ≤ 175 µg/l is correlated with insufficient iodine intake in pregnancy (pregnancy mUIC ≤ 150 μg/l). Twenty-two African countries had SAC mUIC < 175 μg/l, which correlated with insufficient iodine intake during pregnancy (pMUIC < 150 μg/l). However, nine of these twenty-two countries had adequate iodine intake based on SAC mUIC.

**Conclusions:**

There is likely a high prevalence of insufficient iodine intake in pregnancy, including in some African countries classified as having adequate iodine intake in the general population. A SAC mUIC ≤ 175 µg/l predicts insufficient iodine intake among pregnant women in these settings.

More than 89 % of the African population was at risk of insufficient iodine intake before the wide-scale implementation of population-oriented iodine supplementation through food fortification around 1995^(1,2)^. Iodine deficiency to a large extent was a result of low iodine content in soil and groundwater, and a diet rich in goitrogens^(3–5)^. As from 1995, nationwide universal salt iodisation became the main approach of population-based iodine fortification in most African countries^(6)^. Other vehicles of iodine fortification in some African countries include bouillon cubes, canned foodstuffs and other processed foods which are not necessarily regularly consumed^(7,8)^.

Iodine nutritional status in countries in Africa and elsewhere around the world has been monitored using school-age children (SAC) median urinary iodine concentration (mUIC)^(9)^. However, SAC mUIC does not correctly predict iodine nutritional status in pregnancy^(10,11)^. This can be attributed to the pregnancy-induced physiological changes that predispose pregnant women to iodine deficiency through increased thyroid hormone production, increased renal perfusion, increased iodine filtration and urinary iodine excretion in addition to increased transfer of iodine to the fetus^(12)^. In the general population, a mUIC of 100–199 μg/l is considered sufficient nutrition, while a pregnancy median UIC (pMUIC) below 150 μg/l indicates insufficient iodine intake^(12)^.The prevalence of insufficient iodine intake in pregnancy is likely to be high in African countries where a significant proportion of the population has mUIC below 150 μg/l. In such countries, large proportions of women of childbearing age could therefore be at risk of iodine deficiency in pregnancy and associated adverse outcomes such as miscarriage, fetal and childhood growth restriction, still-birth, postpartum thyroiditis, subclinical and overt hypothyroidism, dyslipidaemia, neuro-cognitive and psychomotor deficits^(13–15)^. There is a paucity of data on iodine nutrition status in pregnancy in Africa. Hence, this study was carried out to assess the prevalence of iodine deficiency in pregnancy in Africa using the estimated pMUIC.

## Methods

**Fig. 1:**
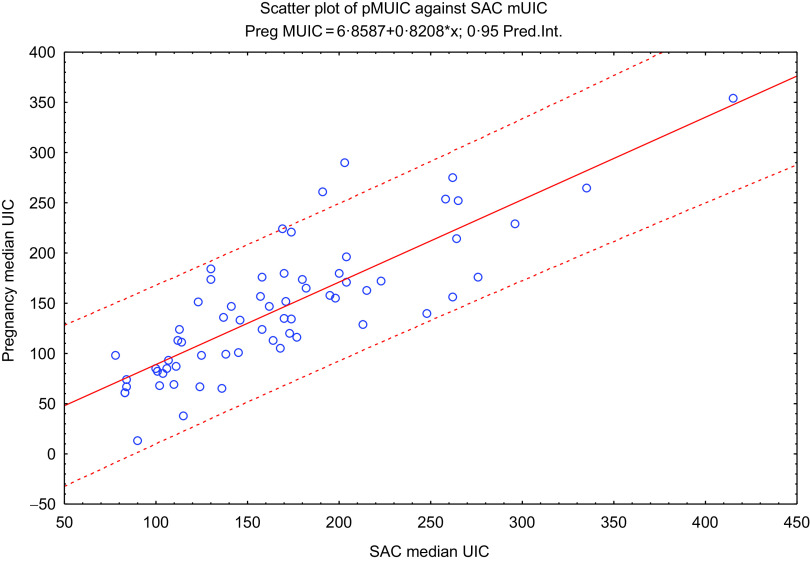
Scatter plot, line of best fit and regression equation of pregnancy median urinary iodine concentration (UIC) against school-age children (SAC) median UIC

We estimated iodine nutrition in pregnancy using the most recent SAC mUIC from the Iodine Global Network (IGN) 2019 score card^(16)^. The IGN publishes and updates the national iodine nutrition status for most countries every 2 years. In the IGN 2017 score card^(17)^, sixty-five countries, including eleven in Africa, had data showing both SAC mUIC and pMUIC. We plotted the SAC mUIC against the pMUIC of these sixty-five countries to depict the linear relationship between the SAC mUIC and the pMUIC (Fig. 1) defined by the following regression equation:

pMUIC = 6·8587 + 0·8208 × SAC mUIC (*r* = 0·8164, *P* <  0·001; *r*^2^ = 0·6665)

Using this regression equation and the most recent SAC mUIC of African countries obtained from the IGN 2019 score card^(16)^, the pMUIC was estimated for all the African countries with recent iodine nutrition survey data.

## Results

Fifty of the fifty-five countries in Africa had data on iodine nutrition status in the general population, estimated mainly through SAC UIC surveys (44/50) carried out between 2002 and 2018, exception for Botswana, Central African Republic, Rwanda and Swaziland where the most recent SAC UIC surveys were conducted between 1993 and 1999. Eleven of these fifty countries also had pMUIC survey data^(16,17)^. Four countries had the iodine nutrition status estimated through surveys involving samples of women of reproductive age (Algeria, Gambia, Madagascar and Sierra Leone), one from a sample of adults (Mauritius) and another from a sample with broad age (Central African Republic). Five countries (Congo, the Comoros, Libya, Sao Tome and Principe, and Seychelles) had no data on iodine nutrition.

Of the fifty countries with UIC survey data available, three had insufficient iodine intake with moderate deficiency (20–49 µg/l); seven had insufficient iodine intake with mild deficiency (50–99 µg/l); eighteen had adequate iodine intake (100–199 µg/l); seventeen had more than adequate iodine intake (200–299 µg/l) exposing susceptible population groups to iodine-induced hyperthyroidism, while five had excessive iodine intake (≥300 µg/l) which increases the risk of autoimmune thyroid disease in addition to iodine induced hyperthyroidism^(18)^ (Table 1).

**Table 1:** School-age children (SAC) median urinary iodine concentration (mUIC), measured and estimated pregnancy mUIC (pMUIC) (μg/l) for African countries

Country	SAC mUIC	*Measured pMUIC	Estimated pMUIC
Northern Africa			
Algeria	241		204·7
Egypt	170	135	146·4
Libya	NA		NA
Mauritania	179		153·8
Morocco	96	31	85·7
Sudan	66		61·0
Tunisia	220		187·4
Western Sahara	NA		NA
Central Africa			
Angola	29		30·7
Cameroon	190		162·8
†CAR	21		24·1
Chad	213		181·7
Congo	NA		NA
DRC	249		211·2
Equatorial-Guinea	564		470
Gabon	196		167·74
Sao Tome & Principe	NA		NA
West Africa			
Benin	318		267·9
Burkina Faso	99	74	88·1
Cabo Verde	115		101·3
Cote d’Ivoire	203		173·5
Gambia	143		124·3
Ghana	130	184	113·6
Guinea	139		121·0
Guinea Bissau	110		97·2
Liberia	244	254	218·6
Mali	69		56·64
Mauritania	179		153·8
Niger	101	82	89·8
Nigeria	130		113·6
Senegal	104	80	92·2
Sierra Leone	203	176	173·8
Togo	171		147·2
Eastern Africa			
Burundi	70		64·3
Comoros	NA		NA
Djibouti	335	265	281·8
Eritrea	175		150
Ethiopia	104		92·2
Kenya	208		177·6
Madagascar	46		44·6
Malawi	269		227·7
Mauritius	160		138·2
Mozambique	60		49·3
†Rwanda	298		251·5
Somalia	417		349
South Sudan	94		84·0
Tanzania	204	171	174·3
Uganda	464		387·7
Southern Africa			
†Botswana	219		186·6
Lesotho	215		183·3
Namibia	216		177·3
†Swaziland	120		105·4
South Africa	215	163	183·3
Zambia	245		208·0
Zimbabwe	220		187·4

NA: data not available.

* Measured pregnancy median UIC (IGN 2017).

† Data collected before 2002.

The African countries with the lowest iodine intake in the general population (moderate iodine deficiency status) were Angola, Central African Republic and Madagascar. Most countries with mild iodine deficiency were from Eastern and Western Africa, while countries with optimal iodine nutrition in the general population were evenly distributed across the northern, eastern and western regions of Africa. All Southern Africa countries apart from Swaziland had more than adequate iodine intake in the general population (SAC mUIC 200–299 µg/l). Benin, Djibouti, Equatorial-Guinea, Somalia and Uganda had excessive iodine intake (SAC mUIC > 300 µg/l), likely attributable to enthusiastic implementation of iodine fortification programmes without regular evaluation^(18)^; and in the case of Somalia and Djibouti, the use of ground water that has high iodine concentration^(5)^.

Of the eleven African countries with pMUIC survey data, Burkina Faso, Egypt, Morocco, Niger and Senegal had inadequate iodine intake during pregnancy. Ghana, Sierra Leone, South Africa and Tanzania had adequate intake, while Djibouti and Liberia had more than enough iodine intake in pregnancy^(17)^.

**Fig. 2:**
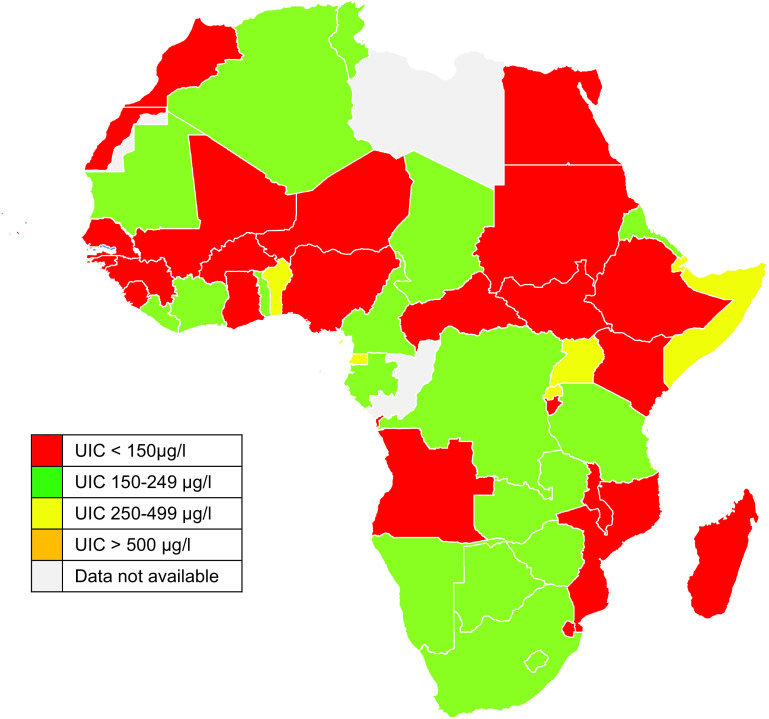
Map of Africa showing country-specific iodine nutrition status during pregnancy (pregnancy median urinary iodine concentration (UIC)) as estimated from school-age children (SAC) median UIC

The pMUIC values calculated in the current study are comparable to findings from population-based surveys from nine countries. The exceptions include Ghana (with sufficient intake) found to have insufficient iodine intake in pregnancy in the current study and Liberia (more than adequate intake) classified as having adequate iodine intake in pregnancy (Fig. 2).

After applying the WHO criteria for monitoring progress towards sustainable IDD elimination where pMUIC < 150 μg/l indicates insufficient iodine intake during pregnancy^(19)^, twenty-two of the forty-four countries with median SAC data available had insufficient iodine nutrition during pregnancy (Fig. 2). Sixteen had adequate and five had more than adequate iodine intake in pregnancy. None of the African countries with available data had pMUIC in the range of excessive iodine intake (pMUIC > 500 μg/l). The current study also found that a cut-off school age mUIC of ≤175 µg/l correlated with insufficient iodine intake in pregnancy (pMUIC ≤ 150 μg/l) (Fig. 1).

Egypt, Gambia, Ghana, Guinea, Guinea-Bissau, Niger, Nigeria, Senegal and Togo are African countries classified as having sufficient iodine intake in the general population (IGN 2019) that in the current study have been found to have insufficient iodine intake in pregnancy (estimated pMUIC < 150 μg/l).

## Discussion

Despite more than 25 years of iodine fortification in Africa, the prevalence of iodine deficiency in pregnancy in Africa appears to have remained high. This high prevalence seems to be masked by the reliance on SAC mUIC for the monitoring of the iodine nutrition status of populations. Although forty out of fifty African countries are currently classified as having adequate or more than adequate iodine intake based on SAC mUIC (IGN 2017 and IGN 2019), the current study estimated that pregnant women in more than half of the forty countries may be prone to insufficient iodine intake. This finding correlates with results from other studies that have shown SAC mUIC to inaccurately reflect the state of iodine nutrition of pregnant women within the same study settings^(10,11,20)^. The observed discrepancy between the SAC mUIC and pMUIC can be attributed to variations in access to iodine-fortified foodstuffs, differences in food choices between school-age children and pregnant women, and the high depletion of iodine stores in pregnancy secondary to increased thyroid hormone production, elevated renal filtration and urinary iodine excretion, and trans-placental transfer of iodine to the fetus^(10–12)^.

If the results of the current study are confirmed by future studies, there will be a need to revisit ongoing iodine fortification strategies, especially in endemic areas for iodine deficiency. While universal salt iodisation is a popular and inexpensive approach suggested by the WHO and ICCIDD, African countries with pMUIC < 150 µg/l could require iodine supplementation during pregnancy, especially those countries with SAC mUIC below 175 µg/l. Oral iodised oil has previously been shown to improve and maintain the level of iodine nutrition within normal limits among adults and children in areas with endemic iodine deficiency, without causing neonatal or maternal hypothyroidism, hyperthyroidism or auto-immune thyroiditis^(21–23)^. This may help mitigate the morbidity that may accrue from widespread insufficient iodine nutrition in pregnancy in Africa.

Since iodine deficiency tends to induce the most adverse effects during pregnancy, infancy and lactation, an appropriate assessment of iodine nutrition would benefit from a direct estimation of the pMUIC as one of the baseline metrics. This can be complemented by assessing the iodine nutrition status of infants, although this may be quite challenging. This could however be achieved through the use of the dry blood spot thyroglobulin test^(24)^ if this becomes readily available for public health use in iodine deficient endemic areas and populations.

Although the current study found that five African countries are likely to have more than adequate iodine intake in pregnancy, the thyroid dysfunction associated with high iodine intake tends to be transient and mild, especially if the increase in iodine intake in formerly iodine-deficient countries was gradual^(18)^. Populations in countries with endemically higher water iodine content such as Djibouti and Somalia tend to be tolerant to high daily iodine intake^(5)^. However, it is recommended that excessive iodine intake should be prevented, especially in countries formerly with chronic iodine deficiency^(25)^.

## Strength and limitations

This study has provided an estimate of the level of iodine nutrition state in pregnancy for most countries in Africa and the minimum SAC mIUC that can be used to predict adequate iodine nutrition status among pregnant women in these settings. The current study design precluded a reliable estimation of the true magnitude of iodine deficiency in pregnancy. However, the data from the current study form a basis for further research to ascertain the true iodine nutrition status among pregnant women in various countries in Africa. While deriving the regression equation for estimating the pMIUC, we assumed that SAC mUIC would correlate with pMIUC. However, this may not always be the case given the different vehicles of iodine fortification around the world and the diverse nutritional practices during pregnancy.

## Conclusion

Insufficient iodine nutrition in pregnancy seems to be prevalent in several African countries, even in countries thought to have sufficient iodine intake in the general population on the basis of SAC mUIC surveys. The SAC mUIC, the current recommended method by WHO to assess iodine nutrition status, seems to conceal insufficient iodine nutrition in pregnancy. Optimum assessment of iodine nutrition status in pregnant women may not be achieved without direct estimation of the pMUIC. Oral iodised oil supplementation could be a possible remedy for insufficient iodine nutrition in pregnancy in countries with a high proportion of the population still prone to moderate-to-severe iodine deficiency despite universal salt iodisation.

## References

[1] Kavishe FP (1996) Can Africa meet the goal of eliminating iodine-deficiency disorders by the year 2000? Food Nutri Bull 17, 262–267.

[2] Joy EJM, Ander EL, Young SD et al. (2014) Dietary mineral supplies in Africa. Physiol Plant 151, 208–229. 24524331 10.1111/ppl.12144 PMC4235459

[3] Taga I, Oumbe VA, Johns R et al. (2008) Youth of west-Cameroon are at high risk of developing IDD due to low dietary iodine and high dietary thiocyanate. Afr Health Sci 8, 180–185. 19357747 PMC2583276

[4] Kassim, IAR, Moloney G, Busili A et al. (2014) Iodine intake in Somalia is excessive and associated with the source of household drinking water. J Nutr 144, 375–381. 24500936 10.3945/jn.113.176693 PMC3927550

[5] Farebrother J, Zimmermann MB, Abdallah F et al. (2018) Effect of excess iodine intake from iodized salt and/or groundwater iodine on thyroid function in nonpregnant and pregnant women, infants, and children: a multicenter study in East Africa. Thyroid 28, 1198–1210. 30019625 10.1089/thy.2018.0234

[6] De Benoist B, McLean E, Andersson M et al. (2008) Iodine deficiency in 2007: global progress since 2003. Food Nutr Bull 29, 195–202. 18947032 10.1177/156482650802900305

[7] Abu BAZ, Oldewage-Theron W & Aryeetey RNO (2018) Risks of excess iodine intake in Ghana: current situation, challenges, and lessons for the future. Ann NY Acad Sci 1446, 117–138. 30489642 10.1111/nyas.13988 PMC6618322

[8] Spohrer R, Knowles J, Jallier V et al. (2015) Estimation of population iodine intake from iodized salt consumed through bouillon seasoning in Senegal. Ann NY Acad Sci 1357, 43–52. 26767583 10.1111/nyas.12963

[9] WHO, UNICEF & ICCIDD (2007) Assessment of Iodine Deficiency Disorders and Monitoring Their Elimination: A Guide for Programme Managers. 3rd ed. Geneva: WHO.

[10] Gowachirapant S, Winichagoon P, Wyss L et al. (2009) Urinary iodine concentrations indicate iodine deficiency in pregnant Thai women but iodine sufficiency in their school-aged children. J Nutr 139, 1169–1172. 19403711 10.3945/jn.108.100438

[11] Wong EM, Sullivan KM, Perrine CG et al. (2011) Comparison of median urinary iodine concentration as an indicator of iodine status among pregnant women, school-age children, and non-pregnant women. Food Nutr Bull 32, 206–212. 22073794 10.1177/156482651103200304

[12] Zantour B, Alaya W, Marmouch, H et al. (2013) Hypothyroidism in pregnancy. In Current Topics in Hypothyroidism with Focus on Development, pp. 29–62 [E Potluková, editor]. Rijeka: In Tech.

[13] Zimmerman MB & Boelaert K (2005) Iodine deficiency and thyroid disorders. Lancet 3, 286–295. 10.1016/S2213-8587(14)70225-6 25591468

[14] De Escobar GM, Obregon MJ & del Rey FE (2007) Iodine deficiency and brain development in the first half of pregnancy. Public Health Nutr 10, 1554–1570. 18053280 10.1017/S1368980007360928

[15] Lee KW, Shin D & Song WO (2016) Low urinary iodine concentrations associated with dyslipidemia in US adults. Nutrients. doi: 10.3390/nu8030171. PMC4808899 26999198

[16] Iodine Global Network (IGN) (2019) *Global Scorecard of Iodine Nutrition in 2019 in the General Population Based on School Age Children (SAC)*. Zurich: IGN.

[17] Iodine Global Network (IGN) (2017) *Global Scorecard of Iodine Nutrition in 2017 in the General Population and in Pregnant Women (PW)*. Zurich: IGN.

[18] Andersson M, Karumbunathan V & Zimmermann MB (2012) Global iodine status in 2011 and trends over the past decade. J Nutr 142, 744–750. 22378324 10.3945/jn.111.149393

[19] De Benoist B, Andersson M, Egli I et al. (2004) Iodine Status Worldwide: WHO Global Database on Iodine Deficiency. Geneva: WHO.

[20] Lazarus JH (2014) Iodine status in Europe in 2014. Eur Thyroid J 3, 3–6. 24847458 10.1159/000358873 PMC4005253

[21] Benmiloud M, Chaouki ML, Gutekunst R et al. (1994) Oral iodized oil for correcting iodine deficiency: optimal dosing and outcome indicator selection. J Clin Endocrinol Metabol 79, 20–24. 10.1210/jcem.79.1.8027227 8027227

[22] Furnee CA, Pfann GA, West CE et al. (1995) New model for describing urinary iodine excretion: its use for comparing different oral preparations of iodized oil. Am J Clin Nutr 61, 1257–1262. 7762527 10.1093/ajcn/61.6.1257

[23] Delange F (1996) Administration of iodized oil during pregnancy: a summary of the published evidence. Bull World Health Organ 74, 101–108. 8653811 PMC2486856

[24] Stinca S, Andersson M, Weibel S et al. (2017) Dried blood spot thyroglobulin as a biomarker of iodine status in pregnant women. J Clin Endocrinol Metabol 102, 23–32. 10.1210/jc.2016-2829 27732337

[25] Laurberg P, Cerqueira C, Ovesen L et al. (2010) Iodine intake as a determinant of thyroid disorders in populations. Best Pract Res Clin Endocrinol Metab 24, 13–27. 20172467 10.1016/j.beem.2009.08.013

